# Progress in recapitulating morphogenesis of blood microvascular structures for microphysiological systems development

**DOI:** 10.1042/BST20240572

**Published:** 2025-07-17

**Authors:** Ana Ximena Monroy-Romero, Mathieu Hautefeuille

**Affiliations:** 1 Programa de Doctorado en Ciencias Biomédicas, Universidad Nacional Autónoma de México, Mexico City, Mexico; 2 Development, Adaptation and Aging (Dev2A, CNRS UMR8263, INSERM U1345), Sorbonne Université, Paris, 75005, France

**Keywords:** microfluidics, microphysiological systems, microvessels, morphogenesis, organ-on-chip, vasculature

## Abstract

Microphysiological systems (MPSs) are complex cell culture platforms, designed to closely replicate the cellular microenvironment of tissues under physiopathological conditions. A critical aspect of these systems is the integration of a vascular network, which facilitates nutrient exchange, supports heterotypic cell interactions, and increases culture viability. A top-down engineering approach, where a prefabricated scaffold is used to introduce endothelial cells, has been widely employed. However, promoting self-organization through a bottom-up paradigm has proven more effective in recapitulating the geometric features of microvasculature, particularly the network nature of it as the capillary diameters. *In vivo* vasculature formation occurs primarily through two self-organization processes: vasculogenesis and angiogenesis. These processes follow a series of co-ordinated and regulated steps, driven by microenvironmental cues such as cell identity and heterogeneity, soluble factor distribution, extracellular matrix composition and mechanics, and flow-induced mechanical strains. By incorporating these parameters into *in vitro* platforms, researchers can develop physiologically relevant vascularized MPS for applications in drug development and disease modeling. This review explores the key mechanisms underlying vascular self-organization and highlights how they are being integrated into tissue-specific MPS platforms to achieve vascularization, which enhances the potential of MPS for studying various physiological and pathological processes.

## Introduction

Microphysiological systems (MPSs) are advanced *in vitro* platforms, designed to culture cells in microenvironments that mimic that of human organs and tissues. Their primary goal is to preserve cell phenotype and recapitulate tissue functions, making them valuable for drug development and basic research [1]. Recently, there has been growing interest in incorporating vasculature into these models, both as standalone systems to study vascular diseases and as integrated components to enhance physiological relevance [2]. By doing so, these models can replicate barrier function, drug transport, and dynamic flow conditions. Moreover, vascular networks can simulate *in vivo* perfusion, facilitating nutrient and oxygen delivery in 3D systems, to prevent necrosis or enabling tissue interconnection for systemic pharmacology studies [3]. While progress has been made using a top-down approach, where hollow extracellular matrix (ECM) scaffolds are populated with endothelial cells (ECs), the resulting geometries and scales often fail to accurately replicate microvasculature due to restrictions from the microfabrication techniques employed [4]. This, in turn, alters the hydrodynamic conditions of the model, particularly the shear stress, a dragging force exerted on the surface of ECs, which they can sense, respond to, and adapt to [5,6]. As an alternative, researchers have explored strategies inspired by vascular morphogenesis, leveraging cell self-organization to achieve more physiologically relevant vascular structures.

In vertebrates, the vascular system develops through tightly regulated mechanisms known as vasculogenesis, the *de novo* formation of blood vessels, and angiogenesis, the growth of new vessels from pre-existing ones [7]. Lumenogenesis and network remodeling are also essential for proper vascular function, ensuring efficient blood flow [8,9]. Together, these processes establish a hierarchical system that prevents pressure drops and flow disturbances: the macrovasculature facilitates the rapid distribution of blood throughout the body, while the microvasculature allows the exchange of molecules such as oxygen, nutrients, and waste products through diffusion within tissues [10]. Beyond its role in transport, the blood microvasculature actively maintains homeostasis by regulating blood flow, immune cell adhesion and infiltration, and reparative processes [11]. However, its dysregulation can contribute to disease onset or progression, for example, by facilitating metastatic cell migration [12] or by impairing regulatory capacity, as seen in portal hypertension associated with hepatic fibrosis [13]. This highlights the importance of developing tissue-specific blood microvasculature models to investigate cellular responses to physiological and pathological stimuli.

The blood microvasculature consists of an organized network of capillaries, ranging from 5 to 10 µm in diameter, connected to arterioles and venules ranging from 10 to 50 µm in diameter [14]. However, the architecture and cellular types of these structures vary depending on the tissue. For instance, hepatic capillaries or sinusoids are highly permeable due to their fenestrated ECs and discontinuous basement membrane. These capillaries are surrounded only by quiescent hepatic stellate cells, which differentiate into myofibroblasts upon activation [15,16]. In contrast, the blood-brain barrier (BBB), characterized by its selective permeability, consists of tightly sealed ECs, a continuous basement membrane, pericytes, and astrocyte end-feet [17]. Regardless of the tissue to be mimicked, engineered microvascular structures must meet essential structural and functional criteria [18]. Structurally, they should consist of an endothelial monolayer with mature tight junctions to replicate barrier functions and must be lumenized to allow perfusion. Functionally, they should regulate permeability and adapt to shear stress [19]. Further refinement of the model would involve incorporating mural cells into the system. Currently, MPSs are being developed focusing on mimicking the sequential steps of *in vivo* vasculogenesis and angiogenesis, seeking to generate perfusable, physiologically relevant microvessels [20–22].

This review aims to serve as a guide to the environmental conditions necessary for generating a vascularized MPS. First, we present *in vivo* vascular morphogenesis, focusing on the key cellular processes involved. Next, we discuss the biochemical and mechanical cues required to replicate these processes *in vitro*. Finally, we highlight some of the current tissue-specific MPS platforms to showcase the development of the field and discuss future directions.

### *In vivo* microvascular morphogenesis

The capillary niche is among the earliest structures formed by the **aggregation** of mesodermal cells from the intermediate layer of the gastrula, giving rise to blood islands through a process mediated by the Flk-1 receptor (VEGFR2) [23]. Cells at the periphery of these aggregates **differentiate** into angioblasts, while those at the core become hematopoietic stem cells [24], a process partly regulated by fibroblast growth factor [25]. Angioblasts then **proliferate** and differentiate into ECs as they are recruited to form multicellular cords, guided by cell–ECM and cell–cell interactions, mediated by integrins and ECM components, as well as adherence junctions such as VE-cadherin [26]. Without a predefined pattern in the ECM, angioblast **migration**, regulated by integrin αVβ3, is essential for forming a network of cords known as the primary capillary plexus, a process termed *de novo* vessel formation or vasculogenesis [27].

After the establishment of the primary plexus, the transition from a 2D-like cord to 3D vessel architecture occurs through lumenogenesis, the hollowing of the multicellular cords that allows blood flow passage [9]. During this process, the cells forming the 2D cords undergo apical-basal polarization with the internal sites of cell–cell contact becoming the apical membrane, towards which negatively charged glycoproteins are directed [28,29]. Due to increased internal pressure, driven by vacuole formation and coalescence on the apical cell surface, the lumen begins to expand [30]. ECs then generate the necessary force to expand the lumen through cytoskeletal complexes, particularly vascular endothelial growth factor (VEGF)- and Rho-associated protein kinase or Rho-associated coiled-coil kinase (ROCK)-mediated actomyosin contractions, allowing intraluminal flow [31]. Moreover, as the lumen expands, tight junction maturation occurs, contributing to the establishment of endothelial barrier function [32]. Tight junctions are essential for regulating paracellular diffusion and maintaining the integrity of the newly formed vessel, with zonula occludens 1 (ZO-1) playing a key role in regulating tension on VE-cadherin-based adherens junctions, which are necessary for lumen maintenance [33].

Once the network allows intraluminal flow, it has to ensure correct efficient tissue perfusion. To achieve this, the vascular network undergoes rearrangements and structural alterations known as remodeling. Specifically, capillary niches formed within each organ independently develop by invading growing tissues through angiogenesis, where vessels extend from pre-existing ones [34]. This process is mediated by the activation of ‘tip’ and ‘stalk’ cells through the VEGF-Dll4-Notch signaling pathway, resulting in branched vessels [35]. When exposed to a VEGF A gradient, often originating from hypoxic tissues, tip cells are activated, extending polarized protrusions where VEGFR2/3 receptors are expressed [36]. This increases VEGF sensitivity and promotes directed migration via ECM degradation through matrix metalloproteinase-2 (MMP2) [37]. Through lateral inhibition, VEGF receptors are down-regulated in neighboring cells, differentiating them to ‘stalk’ cells [38]. These stalk cells then proliferate and elongate the branched structure, forming a new blood vessel where flow is essential for lumen expansion, as observed in zebrafish vasculature [39]. In addition to biochemical gradients, sprouting can also be controlled by the mechanical environment. In quail embryos, sprouts have been shown to form in regions of minimal shear stress and are guided by a pressure gradient from lower to higher pressure points [40]. Other remodeling processes include intussusception, where a vessel splits into two [41]; fusion, where two neighboring vessels merge to form a larger shared lumen [42]; and regression or pruning, where the network is trimmed [43]. These events are mediated by cell proliferation, cell death, cell migration, and ECM production or degradation [7] and have been shown to be regulated by blood flow. In particular, the deletion of mechanosensor Piezo 1 in mice embryos is lethal, impeding vascular remodeling [44]. Furthermore, low shear stress enhances vascular endothelial-cadherin phosphorylation promoting fusion, whereas high shear stress prevents it [45,46]. Similarly, pruning of vessels has been observed in zebrafish brain capillaries when exposed to low shear stress [47].

Further maturation of the microvasculature involves the recruitment of mural cells and the establishment of venule and arteriole phenotypes. Regarding cell recruitment, this process is primarily regulated by platelet-derived growth factor-BB, produced by ECs, and its receptor, platelet-derived growth factor receptor β, which is expressed in mural vascular smooth muscle cells (SMCs) and pericytes (PDGF-BB/PDGFR-β signaling) [48]. Additionally, the transforming growth factor-ß (TGF-β) signaling pathway contributes to mural cell differentiation, recruitment, and stabilization [49], while Notch signaling regulates vascular smooth muscle development [50]. Other factors, such as sphingosine-1-phosphate-1 (S1P-1) and the angiopoietins, have also been implicated in this process [51]. These mural cells contribute to vessel stabilization by promoting the maturation of EC junctions [52] and facilitating the deposition of basement membrane components [53]. Additionally, their contractility is crucial for the regulation of blood flow [54]. Regarding the establishment of venules and arterioles, during the early organization of the capillary plexus, angioblasts begin differentiating into either venous or arterial endothelial phenotypes. This differentiation drives plexus remodeling through EphrinB2/EphB4-mediated cell repulsion, which segregates cells into arterial and venous territories, respectively [55]. As luminal flow is established, cells exposed to higher shear stress further differentiate into an arterial phenotype, while those exposed to lower shear stress develop into a venous phenotype, as observed in chick embryos [56,57].

It is through these morphogenetic steps, driven by co-ordinated cellular processes, such as aggregation, differentiation, proliferation, migration, polarization, and cell death, in which the vascular system is established. Focusing on specific steps of this process has highlighted the importance of ECM composition and mechanics, the presence of soluble factors provided by heterotypic cell populations, and the influence of flow and its corresponding mechanical stimulus in guiding organization. Therefore, these microenvironmental parameters must be incorporated into *in vitro* platforms.

### *In vitro* recapitulation of vascular morphogenesis

**Figure 1:**
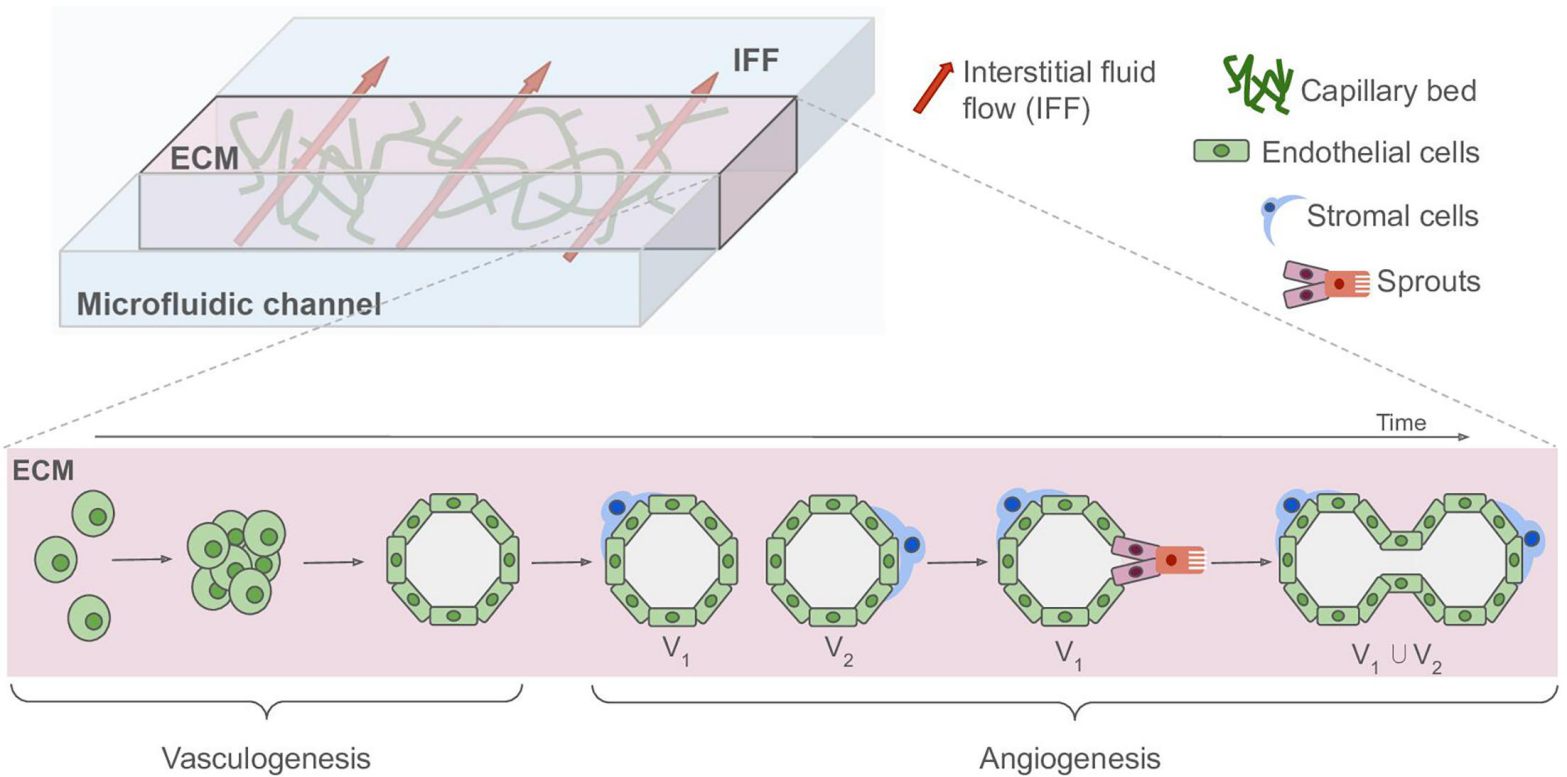
Schematic of a self-organized capillary bed formed through the recapitulation of microvascular morphogenesis. Top: Endothelial cells (ECs), either non-specific or tissue-specific, embedded in an extracellular matrix (ECM) with an established composition, concentration, and stiffness, will organize into a perfusable capillary network. Integration of the ECM with microfluidic channels allows exposure to controlled interstitial and intraluminal flow, promoting vessel maturation. Additional signaling, provided by exogenous growth factors or co-cultured stromal cells, will further enhance vessel integrity. Bottom: Sequential morphogenetic steps by which ECs recapitulate processes such as vasculogenesis and angiogenesis. Scattered cells aggregate and form thin cords through migration and proliferation. These cords then transition into lumenized vessels, driven by interstitial flow, which eventually becomes intraluminal flow, maturing junctions and reducing vessel permeability. These platforms can subsequently be integrated with various parenchymal tissues to further enhance the physiological relevance of the models.

Before generating a model, researchers must first decide on the complexity to achieve (Figure 1). A common starting point is to create a network using only ECs and gradually increase complexity by adding supporting cells. Nevertheless, EC heterogeneity across *in vivo* tissues [58] presents a significant challenge on how to decide the source of the cells. ECs can be sourced from various origins, each with distinct advantages and limitations [59]. Immortalized ECs offer high availability and ease of use, making them suitable for studying environmental factors that drive *in vitro* vascular morphogenesis. However, they often lose phenotype fidelity, leading to responses that may not accurately mimic *in vivo* behavior. In contrast, primary ECs retain tissue-specific characteristics but are limited in availability. Among them, human umbilical vein endothelial cells (HUVECs) are widely used in vascular engineering, with many models optimized for this cell type. Alternatively, ECs derived from human-induced pluripotent stem cells (hiPSC-ECs) offer a versatile option for generating tissue-specific phenotypes through complex differentiation processes, which are crucial for developing physiologically relevant MPS [60]. However, although the majority of differentiation protocols yield hiPSC-ECs that express canonical endothelial markers (CD31, VE-cadherin, and von Willebrand factor), functional assays frequently reveal variations in maturity and functionality. Furthermore, transcriptomic analyses occasionally demonstrate disparities in gene expression profiles associated with vascular identity and function [61]. A recent approach involves the transient reactivation of the embryonic-restricted ETS variant transcription factor 2, which reprograms adult ECs into an adaptive vascular niche. This technique enables ECs to dynamically adjust and integrate with organoids and tumoroids in a tissue-specific manner [62].

To achieve a vascularized and perfused MPS, a microfluidic setup is required. This system consists of a pump, tubing, connectors, and a microfluidic chip [63]. While various commercial microfluidic chips are available, researchers can also fabricate custom designs using microfabrication techniques such as Computer Numerical Control (CNC) machining [64]. This allows for design modifications tailored to specific experimental needs, though it requires access to specialized facilities. For vascular network models, microfluidic chips typically feature three channels separated either by pillars or by a phaseguide structure [65,66]. The ECM is introduced into the central channel, where surface tension, determined by the chip’s geometrical features and the protein gel viscosity, helps confine it within this region. Once polymerized, the ECM remains as a permeable structure. The experimental approach depends on the desired vascularization process. For vasculogenesis models, ECs are mixed with the ECM solution before deposition and gelation. In contrast, to study angiogenesis, cells are introduced only after the ECM has fully polymerized [67].

ECs cultured in collagen I or fibrin gels, reconstituted from fibrinogen and thrombin, can self-organize into vascular networks. When aiming to recapitulate vasculogenesis-like organization, network architecture, including vessel length and width, is influenced by scaffold stiffness and permeability, both of which correlate with protein concentration [68,69]. Common concentrations range from 2 to 4 mg/ml for collagen I and 2.5 to 6 mg/ml for fibrin. For angiogenesis-like models, the gel serves as a physical barrier that supports growth factor gradient formation and mechanically stabilizes endothelial sprouts. The composition of these gradients significantly affects sprout maturity. For example, sprouts generated with only a VEGF gradient tend to be shorter and more permeable compared with those induced by a combination of VEGF, S1P, and PMA [70]. Similar to vasculogenesis models, matrix concentration and density affect invasion depth and sprout diameter. Higher matrix density enhances EC invasion while reducing migration speed [71]. ECs sense and respond to mechanical cues through various signaling pathways, particularly the Hippo pathway, where the transcriptional cofactors YAP/TAZ play a central role [72]. A feedback loop within this pathway limits cytoskeletal and focal adhesion maturation, thereby regulating EC mobility and organization [73]. This process is mediated in part by integrin αVβ3 and MMP2 activity [74].

Lumen formation in EC-embedded gels depends on the presence of interstitial fluid flow, which enhances network formation and accelerates organization by promoting ECM remodeling through MMP-2 up-regulation [75]. Once the lumen has opened, intraluminal flow can be introduced using microfluidic pumps interfaced with chips containing the pre-formed vascular network or by rocking the chip with reservoirs [76]. Ensuring physiologically relevant flow exposure is crucial, as optimal flow conditions vary by tissue and depend on chip design and pump selection. Applying luminal wall shear stress (WSS) within fibrin-based capillary networks stabilizes vessel diameter and significantly alters gene expression, enhancing barrier function, reducing reactive oxygen species, and down-regulating anti-angiogenic cytokines [77]. WSS also regulates vascular integrity by activating the NOTCH1 receptor, which promotes adherens junction assembly. In contrast, static conditions often result in leaky structures [78]. Perfused microfabricated vessels effectively recapitulate vascular permeability, as demonstrated by the distribution of soluble molecules [79], a key feature in preclinical models evaluating soluble therapeutic agents [80]. Additionally, microvessel models facilitate cancer-related biophysical studies, enabling *in situ* tracking of cellular arrest and extravasation [81] and investigating how transmural flow in leaky vessels up-regulates PD-L1 expression [82].

Supporting cells can be incorporated into vascular models through co-culture with tissue-specific pericytes, SMCs, stromal cells, fibroblasts, and dental pulp stem cells (DPSCs). DPSCs, in particular, promote vascular morphogenesis by releasing VEGF, a key factor in vasculogenesis and angiogenesis [83]. Tissue-specific stromal cells can also influence EC phenotype, guiding naïve ECs to express markers more closely resembling *in situ* tissue ECs [84], which, in turn, affects vessel dimensions and perfusability [85]. Pericytes contribute to vascular stabilization by expressing NOTCH3, which interacts with EC-expressed NOTCH1 to regulate endothelial junction integrity through Notch signaling [86]. Fibroblasts are increasingly used in vasculogenesis models as they enhance vascular function by stiffening the scaffold via a YAP1-mediated, Hippo pathway-dependent process [87]. However, when fibroblasts are not part of the native microvascular environment, they can be selectively ablated through controlled apoptosis [88], as their supportive role is primarily required during early vascular organization. Additionally, fibroblast incorporation has helped to identify mechanics as an independent driver of endothelial self-organization. Cancer-associated fibroblasts, for instance, promote angiogenesis by increasing mechanical strain, even in conditions of reduced soluble factor diffusion [89]. Furthermore, a human-heart MPS has enabled us to understand that primitive macrophages prime stromal cells for long-term cardiac vascular development [90]. This last work showed the importance of using such immune cells in complex tissue-specific vascular MPS, as it revealed how macrophages participate very strongly in the establishment of stable, functional perfusable microvessel architecture. Thanks to relevant and pivotal cell–cell cross-talk, this looks promising and is being applied to other tissues.

The incorporation of one or more of these parameters into *in vitro* models has enabled the development of various vascularized tissue platforms. Additionally, there is growing interest in culturing patient-derived cells to generate more physiologically relevant models, as we will describe next. These platforms have successfully recapitulated *in vivo*-like responses, making them promising for further advancement.

### Tissue-specific vascularized microphysiological systems

Through the generation of vascularized models, recapitulation of responses *in vivo* has been observed. For instance, mammary microvessels, established from primary breast ECs and fibroblasts, exhibited phase-dependent vascular remodeling and barrier function changes in response to menstrual cycle hormones, a response that was not observed in HUVEC-based vessels [91]. Similarly, fetoplacental vasculogenesis, crucial in the interchange of molecules between the placenta and fetus, has been replicated in a model incorporating human placenta terminal villi, fetal mesenchyme, vascular endothelium, fibroblasts, and pericytes. In this model, increased shear stress enhanced fetal capillary barrier function, suggesting that alterations in flow could be linked to gestational disorders [92].

Vascularized MPS devices also aim to study the effect of particular stimuli in physiopathological environments, for example, the stiffness of the tissues. An increase in stiffness has been observed in both aging tissues [93] and fibrotic organs [94]. Using a gradual stiffening gel where a vascular network from EC-iPS was initially formed under soft conditions showed that increased stiffness disrupted the network due to β-catenin dissociation. Inhibiting cell contractility partially rescued the juvenile EC phenotype in this model, shedding light on a potential therapeutic target [95]. By modulating the stiffness of a fibrin gel with the addition of stromal cells to lung and cardiac-specific hiPSC-EC networks, the role of TGF-β, a key player in fibrosis, was investigated. The impact was found to be more severe under soft conditions where stromal cells were absent [96]. Matrix stiffness also plays an important role in ECs’ traction behavior, as increased traction force and energy usage were recently associated with higher substrate stiffness [97].

These models are also intended for drug testing, with key targets like the BBB, which regulates the central nervous system homeostasis. A fibrin model incorporating hiPSC-ECs, brain pericytes, and astrocytes achieved permeability values close to *in vivo* measurements in rat brains [98]. This model enabled the rapid analysis of polymer nanoparticle permeability, serving as a drug transport assay in a physiologically relevant human BBB setup, thereby providing a promising preclinical model [99]. Another significant model is the inner blood-retinal barrier, recapitulated using human retinal microvascular ECs, human retinal pericytes, and human retinal astrocytes. This model replicates the pathophysiological phenotypes observed in diabetic retinopathy, providing a platform for preclinical testing of pericyte-EC stabilizing strategies [100].

By recapitulating vascular morphogenesis, epithelial and cancerous spheroids can now be vascularized and perfused, enabling complex multicellular interactions, including with immune cells. The pancreas has been modeled by incorporating isolated human islets within a 3D matrix with perfusable vessels, demonstrating long-term viability, maintaining robust glucose-stimulated insulin responses, and allowing for the assessment of islet–immune cell interactions, crucial for studying diabetes pathogenesis [101]. Regarding the tumoral microenvironment, monocyte extravasation and differentiation into macrophages in breast cancer and melanoma tumors has been studied using these vascularized platforms [102]. However, changes in vessel density and barrier function, compared with the bed of perfusable vessels, have been reported in vessels surrounding adenocarcinoma human alveolar basal epithelial cells [103], but improvement was achieved by adding fibroblasts to tumor spheroids, which facilitated immunotherapy evaluation using chimeric antigen receptor T cells [104]. Additionally, patient-derived colorectal cancer cells can now be used to generate these models, moving toward the goals of personalized medicine [105].

The recapitulation of microvascular morphogenesis has permitted the development of more sophisticated and accurate models, providing valuable insights into physiological and pathological processes and enhancing the potential for therapeutic development and personalized medicine. However, lumenized endothelial structures have also been formed through mechanisms that have not yet been observed *in vivo*, like folding [106]. Therefore, the bottom-up engineering approach not only recapitulates *in vivo* events of vascular development but also raises the question of whether phenomena seen in *in vitro* models could similarly occur in developmental processes (Table 1).

**Table 1:** Key factors in vasculogenesis and their recapitulation in MPS. Summary of essential elements involved in vascular morphogenesis, their functions *in vivo*, and the strategies used to replicate these processes in microphysiological systems.

Element in microvasculature morphogenesis	Functions *in vivo* vasculature	Impact and strategies in MPS
**Precursor cells and endothelial cells (ECs**)	Form the lining of vasculature through aggregation, differentiation, proliferation, migration, and polarization [23–27].	Select the source of ECs: immortalized cell lines [59], primary ECs [59], hiPSC-EC [60], and reprogrammed adult EC [62].
**Extracellular matrix (ECM**)	Provides structural support and biochemical signals [26].	Collagen and fibrin hydrogels [68,69].
**Shear stress (Blood flow**)	Serves as a transport system of nutrients and waste, exposes cells to shear stress [5,6].	Microfluidic systems with controlled flow, peristaltic pumps, and microfluidic chips [63].
**Biochemical gradients (VEGF, TGF-β, Notch, etc**.)	Direct migration, proliferation, vascular remodeling, and mural cell recruitment [34–36,48–51].	Establishment of gradients within a porous gel [70].
**Supporting neighboring cells** (**fibroblasts, pericytes, mural cells, and macrophages**)	Stabilize capillaries and regulate permeability [53–55].	Co-culture with fibroblasts [87,89], pericytes [86], DPSCs [83], or macrophages [90].

DPSCs, dental pulp stem cells. hiPSC, human-induced pluripotent stem cell.

### Challenges of vascularized MPS

Despite significant advancements, several challenges remain in developing fully functional vascularized MPS. One major issue is the heterogeneity of EC populations, as faithfully recapitulating tissue-specific endothelial phenotypes is essential for physiological accuracy. Additionally, ensuring the long-term stability of vascular networks over extended culture periods remains a crucial objective to enhance the reliability of these models. Another challenge is the integration of vascularized compartments with other tissue types, as multi-tissue platforms are necessary to better replicate the complexity of human physiology. Finally, rigorous validation against *in vivo* data is required to confirm the translational potential of MPS and ensure their relevance in biomedical applications. Addressing these challenges will be essential for advancing the utility of vascularized MPS in both fundamental research and clinical settings.

**Perspectives**

- Studying the mechanisms that guide microvasculature morphogenesis allows the identification of critical biomechanical cues (specification), which need to be integrated into *in vitro* models. These models, engineered to integrate these adequate cues, can enable the recapitulation of cell self-organization in vascular structures that then mimic the vasculature of specific tissues, necessary for cellular communication in pathophysiological processes and greatly improve the fidelity of the models.
- Currently, strategies have been developed to obtain tissue-specific endothelial cells (ECs), and protocols have been established to generate stable vascular networks through the fine-tuning of pivotal mechanical microenvironment parameters, such as substrate stiffness and shear stress from interstitial and intraluminal flow. All these efforts aim to create relevant platforms that can serve as preclinical models for various diseases after biological validation.
- The future aim is to integrate various models to build increasingly faithful representations of a complete organism, rather than studying tissues in an individual manner. Additionally, the identity of ECs in these models must be thoroughly verified, as the heterogeneity within single tissues must be accounted for. The exact ontogenesis of capillary formation still remains unclear. For example, the influence of lymphatics on blood vessel growth and health must be studied, especially their regulation of the overall interstitial fluid flow within the tissue.

## Abbreviations

BBB: blood-brain barrier
DPSCs: dental pulp stem cells
EC: endothelial cell
ECM: extracellular matrix
HUVECs: human umbilical vein endothelial cells
MMP2: matrix metalloproteinase-2
MPS: microphysiological systems
ROCK: Rho-associated coiled-coil kinase
SMCs: smooth muscle cells
S1P: sphingosine-1-phosphate
TGF-ß: transforming growth factor-ß
VEGF: vascular endothelial growth factor
WSS: wall shear stress
ZO-1: zonula occludens 1
hiPSC: human-induced pluripotent stem cell
